# The efficacy and safety of medical thoracoscopy compared with tube thoracostomy in the treatment of complicated parapneumonic effusion and empyema: A retrospective single-center study

**DOI:** 10.1097/MD.0000000000050048

**Published:** 2026-08-14

**Authors:** Yanchao Liu, Yaojie Wang, Xin Liu, Zheng Duan

**Affiliations:** a Department of Respiratory and Critical Care Medicine, The Second Hospital of Hebei Medical University, Shijiazhuang, Hebei, P.R. China; b Second Department of Respiratory and Critical Care Medicine, Cangzhou Central Hospital, Cangzhou, Hebei, P.R. China.

**Keywords:** complicated parapneumonic effusion, empyema, hospitalization time, medical thoracoscopy, tube thoracostomy

## Abstract

To evaluate the efficacy and safety of medical thoracoscopy (MT) compared with tube thoracostomy (TT) for complicated parapneumonic effusion (CPPE) and empyema. We retrospectively analyzed 79 adults with stage 5 CPPE or empyema treated from May 2018 to January 2024. Patients were non-blindly assigned to an MT group (n = 25) or TT group (n = 54) according to attending physician discretion, imaging findings, clinical status, procedural suitability, and physician expertise. Baseline characteristics, pleural fluid biochemistry, clinical improvement, treatment failure, in-hospital mortality, total pre-intervention and post-intervention hospitalization, pleural fluid drainage volume, and postoperative complications were compared. Clinical improvement required symptom resolution plus radiographic reduction without further invasive intervention; treatment failure included persistent sepsis, additional intervention, or pleural-infection-related death. Median total hospitalization was 17 days (inerquartile range [IQR], 13–22) in the TT group and 20 days (IQR, 16–25) in the MT group, without a significant difference (*P* > .05). The MT group had more loculation/septation (92.0% vs 68.5%, *P* = .023) and longer pre-intervention hospitalization (7 days, IQR 5–11 vs 2 days, IQR 1–3; *P* < .05), suggesting more complex disease and delayed escalation. Post-intervention hospitalization was shorter after MT (10 days, IQR 6–17) than TT (15 days, IQR 11–20) (*P* < .05). Pleural fluid drainage volume was greater after MT (1210 mL, IQR 475–2300) than TT (600 mL, IQR 245–1212) (*P* < .05). Clinical improvement, treatment failure, adverse reactions, and mortality did not differ significantly between groups (*P* > .05). The overall adverse reaction rate was 16.0% after MT and 3.6% after TT (*P* = .076). MT appears feasible and safe for selected patients with CPPE and empyema. It was associated with greater pleural drainage and shorter post-intervention hospitalization than TT, despite use in patients with more complex disease. However, because this retrospective study involved non-blinded treatment allocation, selection bias, baseline imbalance, and a small sample, the findings are hypothesis-generating rather than definitive. MT did not show a statistically significant effect on in-hospital mortality. Prospective randomized studies are needed to define its optimal timing and role in pleural infection management.

## 1. Introduction

Parapneumonic pleural effusion (PPE) refers to pleural effusion associated with bacterial pneumonia, lung abscess, or bronchiectasis with infection.^[1]^ Studies show that 20% to 50% of patients with community-acquired pneumonia have evidence of PPE,^[2]^ and approximately 6% progress to complicated PPE (CPPE) or empyema.^[3]^ PPE is associated with increased mortality and hospital costs.^[4–6]^ Various studies report mortality rates as high as 30% for patients with PPE and empyema.^[7–9]^ CPPE increases the risk of death in pneumonia patients by 3 to 6 times.^[10]^

Pleural effusions secondary to pneumonia are classified according to Light’s criteria based on pleural fluid biochemistry and microbiology.^[11]^ Simple parapneumonic effusions are free-flowing and sterile, with pH > 7.20, glucose >40 mg/dL, and lactate dehydrogenase < 1000 IU/L. Complicated parapneumonic effusions (stage 5) are defined by the presence of at least one of the following: pleural fluid pH < 7.0, glucose <40 mg/dL (2.2 mmol/L), positive Gram stain or culture, or the presence of loculations/septations on imaging. Empyema refers to the presence of frankly purulent pleural fluid.^[11]^ Progression from a simple parapneumonic effusion to CPPE or empyema is more likely in patients with delayed or inadequate antibiotic treatment, virulent or polymicrobial infection, impaired host immunity, diabetes mellitus, malignancy, chronic kidney or liver disease, and other comorbid conditions that reduce infection clearance. Early diagnosis, appropriate antibiotic therapy, and adequate drainage are crucial for reducing the morbidity and mortality of PPE.^[12]^ In patients with significant residual pleural effusion, there is a risk of developing chronic pleural inflammation, pleural thickening, and restrictive ventilatory dysfunction.^[13]^

Primary treatments for PPE include antibiotics and thoracic drainage. Tube thoracostomy (TT) is a bedside or image-guided procedure in which a chest tube is inserted into the pleural cavity and connected to a closed drainage system to evacuate infected pleural fluid. Local management strategies for pleural infection also include medical thoracoscopy (MT), indwelling pleural catheter drainage, and intrapleural fibrinolytic therapy, including intrapleural streptokinase evaluated in Multicenter Intrapleural Sepsis 1.^[14]^ Recent advances include the use of video-assisted thoracoscopic surgery (VATS) and intrapleural enzyme therapy, as discussed in recent reviews and evaluated in the Multicenter Intrapleural Sepsis-3 trial.^[11,15]^ However, drainage fails in approximately 30% of patients due to loculation and increased viscosity of the pleural fluid, and about 20% require surgical intervention for adequate treatment of pleural infection.^[16]^ Intrapleural therapy combining tissue plasminogen activator and recombinant human deoxyribonuclease has been shown to improve radiographic outcomes in PPE patients, but this approach is costly and requires prolonged hospitalization (10–14 days).^[17]^ International guidelines suggest surgical thoracoscopy or open thoracotomy when medical therapy and chest tube drainage fail, or when PPE progresses to organized empyema with extensive pleural fibrosis.^[5,18]^ However, these surgical procedures require general anesthesia and are expensive, limiting their applicability in frail or high-risk patients.

Medical thoracoscopy, performed under local anesthesia with conscious sedation, is a minimally invasive endoscopic technique in which a thoracoscope is inserted through a small chest-wall incision to directly visualize the pleural cavity and perform diagnostic or therapeutic interventions. It is used for diagnosing pleural effusions of unknown origin, suspected malignancies, pleurodesis with talc for pneumothorax, and other conditions.^[19–21]^ Compared to conventional medical therapy, it offers several advantages, particularly in cases of loculated effusions. MT allows mechanical breakdown of fibrin adhesions, facilitates direct visualization for optimal chest tube placement, and enables pleural biopsy.^[21]^ It is also suitable for patients unfit for general anesthesia due to its feasibility under local anesthesia with moderate sedation, making it applicable for frail or high-risk surgical patients. Potential limitations and complications include lung injury, bleeding, subcutaneous emphysema, re-expansion pulmonary edema, hypotension, incomplete adhesiolysis in organized empyema, and the need for conversion to VATS or thoracotomy in selected cases.

Several studies have demonstrated the role of MT in treating CPPE and empyema.^[3]^ However, despite these promising results, a specific knowledge gap remains: there is a lack of direct comparative studies evaluating MT versus conventional TT in real-world clinical practice. Furthermore, the optimal timing for MT intervention and its impact on clinically relevant outcomes such as hospitalization duration have not been clearly established. Recent reviews have summarized the evolving landscape of pleural infection management.^[22]^ Due to the absence of large-scale prospective randomized controlled trial evidence,^[5,19]^ there is no definitive consensus on the role of MT in managing PPE and empyema, and current guidelines do not include MT as a standard recommendation.

Therefore, this retrospective study aimed to compare the efficacy and safety of medical thoracoscopy versus tube thoracostomy in patients with CPPE and empyema. Specifically, we evaluated clinical outcomes including treatment success rates, hospital mortality, length of hospital stay (total, pre-intervention, and post-intervention), volume of pleural fluid drained, and procedure-related complications. By providing direct comparative data, we seek to clarify the potential benefits of MT and inform clinical decision-making in the management of pleural infection.

## 2. Materials and methods

### 2.1. Study design and patient selection

This retrospective study enrolled patients with stage 5 complicated parapneumonic effusion (CPPE) or empyema hospitalized in the Department of Respiratory and Critical Care Medicine at The Second Hospital of Hebei Medical University between May 2018 and January 2024. Staging of parapneumonic effusion was based on Light’s criteria.^[11]^

**Figure 1.**
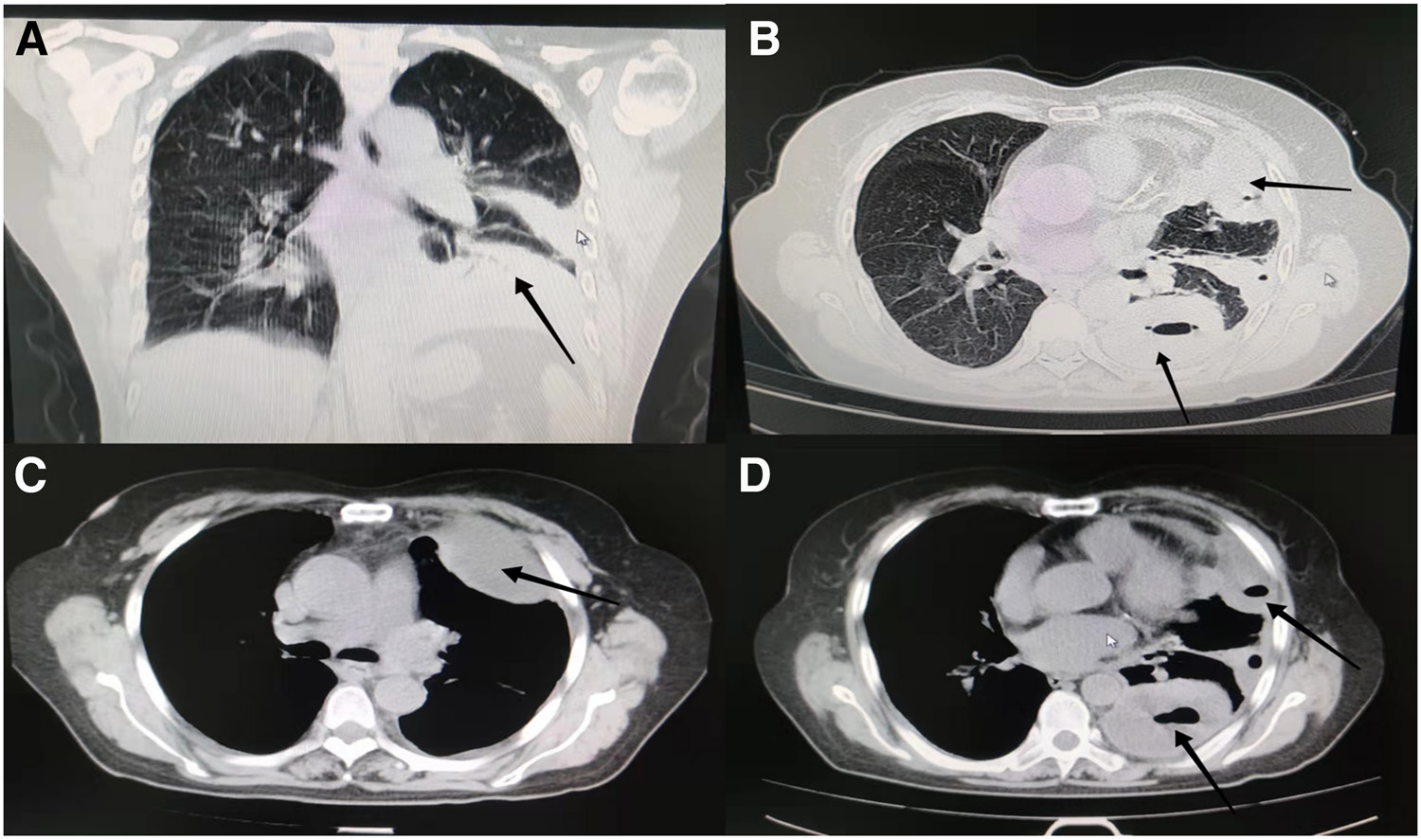
Imaging findings of loculated pleural effusion. (A) Chest radiograph shows a left-sided loculated pleural effusion (black arrows). (B) Chest CT lung window showing empyema (black arrows). (C and D) Mediastinal window shows a left-sided loculated pleural effusion (black arrows). CT = computed tomography.

Inclusion criteria were: stage 5 PPE: pleural fluid pH < 7.0; and/or pleural fluid glucose <40 mg/dL (2.2 mmol/L); and/or positive Gram stain or culture of pleural fluid; loculated/septated pleural effusion on chest computed tomography (CT) or ultrasound (Fig. 1); and empyema: purulent appearance of pleural fluid, with or without loculation.

Exclusion criteria included: age <18 years, malignancy, tuberculous pleural effusion, chronic pleural effusion (present for >3 months), and patients who did not undergo diagnostic thoracentesis.

All enrolled patients underwent diagnostic thoracentesis after admission following chest CT and thoracic ultrasound to characterize the pleural effusion and received appropriate intravenous antibiotic therapy based on local guidelines and culture results when available.

Treatment allocation: the decision to perform MT versus TT was non-randomized and non-blinded and was made at the discretion of the attending physician based on a combination of factors including: imaging characteristics (presence of multiloculation/septation, estimated fluid volume, and evidence of organized pleural thickening); clinical status (severity of illness, response to initial antibiotic therapy, persistent fever, sepsis, or clinical deterioration); patient suitability for the procedure (performance status, comorbidities, and ability to tolerate conscious sedation); and physician preference and expertise. Generally, patients with more complex loculated effusions, suspected organized empyema, persistent fever or inflammatory response despite antibiotics, loculated pockets on imaging, inadequate drainage after initial conservative therapy, or unstable clinical status requiring stabilization before intervention were more likely to be referred for MT. This non-randomized and non-blinded allocation introduces potential selection bias, which we have acknowledged as a study limitation.

Cross-over cases: among patients initially treated with TT, those who failed to improve after 48 to 72 hours of drainage (persistent fever, lack of radiographic improvement, or clinical deterioration) were considered for rescue MT at the physician’s discretion. In the primary analysis, these patients were analyzed according to the intention-to-treat principle, remaining in the TT group. A sensitivity analysis excluding these patients was also performed and did not materially alter the results (data not shown).

Patients were divided into 2 groups based on the initial treatment received: an MT group (n = 25) and a TT group (n = 54).

### 2.2. Procedures

**Figure 2.**
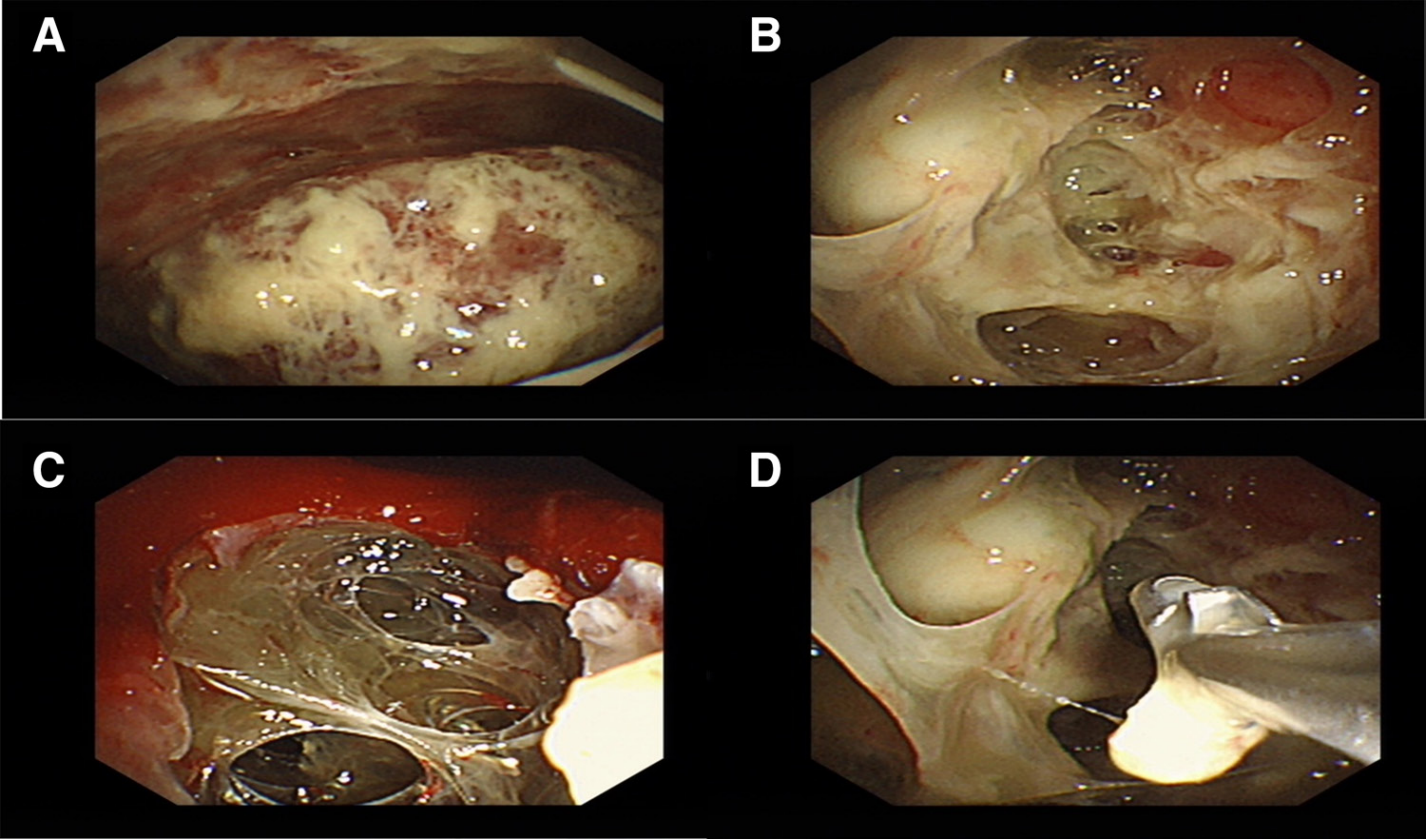
Findings of CPPE and empyema under medical thoracoscopy. (A and B) A large amount of white purulent material in the pleural cavity. (C) Pleural adhesion forming loculated effusion. (D) Video-assisted thoracoscopic forceps clearing adhesions. CPPE = complicated parapneumonic effusion.

MT group: medical thoracoscopy was performed under local anesthesia with intravenous sedation (conscious sedation using midazolam and fentanyl). Patients selected for MT often required a longer pre-interventional period because of greater disease complexity, unstable clinical status, persistent infection, and the need for initial antibiotic therapy and medical stabilization before invasive intervention. During MT, the patient was placed in the lateral decubitus position with the affected side up. After sterilization and draping, local anesthesia (2% lidocaine, 10–20 mL) was administered. A 1 to 2 cm skin incision was made along the intercostal space, subcutaneous tissues were bluntly dissected to the parietal pleura, and an 8 to 10 mm trocar sheath was inserted. A rigid or semi-rigid 7-mm thoracoscope was introduced into the pleural cavity. Pleural fluid was slowly aspirated, air entry was allowed intermittently to improve visualization and lung collapse, adhesions and loculations were cleared with biopsy forceps, fibrinous/purulent material was aspirated, and the pleural cavity was thoroughly irrigated with warm saline. Pleural biopsies were taken from suspicious parietal pleural areas when present. A chest tube (24–28 Fr) was placed postoperatively for continuous closed drainage at −20 cm H_2_O suction. Chest CT or ultrasound was performed 24 hours post-operation to assess residual fluid. The chest tube was removed when there was no significant residual pleural effusion (<200 mL on ultrasound), drainage was <75 mL/d, and clinical symptoms had significantly improved (Fig. 2).

TT group: ultrasound-guided thoracentesis and chest tube placement (24–28 Fr) were performed for closed drainage. The puncture site was selected according to ultrasound findings; local anesthesia was administered, pleural fluid was aspirated to confirm location, and the tube was inserted into the pleural cavity using standard sterile technique and connected to a closed drainage system. Some patients received intrapleural saline irrigation (100–200 mL normal saline twice daily) at the physician’s discretion, and some received intrapleural urokinase therapy (100,000 IU daily for 3 days) when loculation was present and drainage was inadequate. Chest CT or ultrasound was repeated every 3 to 5 days to assess response. The chest tube was removed when clinical symptoms significantly improved and drainage was <75 mL/day.

Data collected included general information, clinical manifestations, laboratory findings, length of hospital stay, interventions, clinical outcomes, and complications.

### 2.3. Outcome definitions

Primary outcomes were defined as follows:

Clinical improvement was defined as a composite endpoint requiring ALL of the following: resolution of fever (temperature <37.5°C for ≥48 hours) and other infection-related symptoms (chest pain, dyspnea, and cough); radiographic evidence of pleural effusion reduction or resolution (≥50% reduction in effusion size on chest CT or ultrasound, assessed by a radiologist unaware of the study objectives); and no requirement for additional invasive interventions beyond the initial procedure (including repeat thoracoscopy, VATS, open thoracotomy, or image-guided drainage).

Treatment failure was defined as the occurrence of any of the following: persistent sepsis or clinical deterioration despite drainage (persistent fever, leukocytosis, or hemodynamic instability requiring vasopressors); the requirement for additional invasive interventions (as defined above); and in-hospital death attributable to pleural infection (determined by independent review of clinical records by 2 physicians).

Post-intervention hospital stay was defined as the number of days from the date of intervention (MT or TT) to hospital discharge.

Pre-intervention hospital stay was defined as the number of days from hospital admission to the date of intervention.

Total hospital stay was defined as the number of days from hospital admission to discharge.

Total volume of pleural fluid drained was defined as the cumulative volume of fluid collected via chest tube from placement until removal.

Due to the retrospective design, outcome assessors were not blinded to the intervention type, as this information was documented in patient records. This represents a potential source of bias and has been acknowledged as a study limitation.

### 2.4. Ethics statement

This study was performed in accordance with the Declaration of Helsinki and was approved by the Research Ethics Committee of the Second Hospital of Hebei Medical University (approval number: 2020-R701-R01). All adult participants provided written informed consent to participate in this study.

### 2.5. Statistical analysis

Data were analyzed using SPSS version 26.0 (IBM Corp.).

Normality assessment: the Shapiro–Wilk test was used to assess the normality of continuous variable distributions. Variables with a normal distribution (age, peripheral blood white blood cell count, and neutrophil percentage) were presented as mean ± standard deviation and compared using the independent samples *t* test. Variables with a non-normal distribution (all other continuous outcomes, including hospitalization times, drainage volumes, and laboratory parameters) were presented as median with interquartile range (IQR) and compared using the Mann–Whitney *U* test.

Categorical data were described as frequency (percentage) and analyzed using the chi-square test or Fisher exact test, as appropriate.

Confidence intervals (CIs): for all key outcome measures, 95% CIs for between-group differences were calculated. For median differences, the Hodges–Lehmann estimator was used. For proportion differences, the Newcombe-Wilson method was applied.

Adjustment for confounders: We acknowledge that baseline imbalances exist between groups, particularly in the rate of loculated effusions and pre-intervention hospital stay. Given the retrospective and exploratory nature of this study, as well as the relatively small sample size, which limits statistical power for multivariate modeling, we have chosen to present only unadjusted comparisons in the primary analysis. This limitation is explicitly acknowledged in the Discussion section, and we recommend that future prospective studies with larger sample sizes incorporate multivariate analyses to confirm our findings.

A *P* value <.05 was considered statistically significant. All tests were 2-tailed.

## 3. Results

### 3.1. General characteristics

A total of 79 patients with stage 5 CPPE or empyema were enrolled in this study: 54 patients in the TT group and 25 patients in the MT group.

The TT group had a median age of 63 years (IQR, 53–74), with 42 males (77.8%) and 12 females (22.2%). The MT group had a median age of 61 years (IQR, 55–68), with 16 males (64%) and 9 females (36%). There were no significant differences in age or sex between the groups (*P* > .05).

No significant differences were found between groups in clinical symptoms, organ failure, need for mechanical ventilation, location of pleural effusion, or proportion receiving intrapleural urokinase (*P* > .05). However, notable baseline differences were observed. The rate of loculation/septation was significantly higher in the MT group compared to the TT group (92.0% vs 68.5%, *P* = .023). Additionally, the median pre-intervention hospital stay was significantly longer in the MT group (7 days, IQR 5–11) than in the TT group (2 days, IQR 1–3) (*P* < .001). These differences imply that patients in the MT group had more advanced or complex pleural disease at presentation, which is important to consider when interpreting subsequent outcomes (see Table 1).

**Table 1:** Comparison of general characteristics between groups.

Item	Tube thoracostomy group (n = 54)	Medical thoracoscopy group (n = 25)	Statistic	*P* value
Age (yr)	63 (53–74)*	61 (55–68)*	Z = -0.855	.393
Male	42 (77.8%)	16 (64.0%)	χ^2^ = 1.662	.197
Symptoms				
Fever	38 (70.4%)	17 (68.0%)	χ^2^ = 0.045	.831
Dyspnea	34 (63.0%)	17 (68.0%)	χ^2^ = 0.189	.663
Chest pain	24 (44.4%)	10 (40.0%)	χ^2^ = 0.138	.711
Cough/sputum	20 (37.0%)	12 (48.0%)	χ^2^ = 0.852	.356
Organ failure				
Any organ failure	23 (42.6%)	11 (44.0%)	χ^2^ = 0.437	.509
Respiratory failure	23 (42.6%)	11 (44.0%)	χ^2^ = 0.437	.509
Multiple organ failure	2 (3.7%)	2 (8.0%)	–	.587
Mechanical ventilation	7 (13.0%)	6 (24.0%)	χ^2^ = 1.514	.219
Pleural loculation/sepration	37 (68.5%)	23 (92.0%)	χ^2^ = 5.158	.023*
Empyema location			χ^2^ = 0.329	.567
Unilateral	40 (74.1%)	20 (80.0%)		
Bilateral	14 (25.9%)	5 (20.0%)		
Use of urokinase	12 (22.2%)	4 (16.0%)	χ^2^ = 0.410	.522

* Median (IQR).

* *P* < .05.

### 3.2. Laboratory findings

There were no significant differences between the 2 groups in peripheral blood white blood cell count, neutrophil percentage, erythrocyte sedimentation rate, high-sensitivity C-reactive protein, or in pleural fluid parameters including total leukocyte count, monocyte percentage, neutrophil percentage, lactate dehydrogenase, albumin, and glucose levels (all *P* > .05).

The pleural fluid culture submission rate was significantly higher in the TT group (83.3% vs 60.0%, *P* = .024). This difference may reflect clinical workflow: TT was primarily performed as an initial drainage procedure, during which pleural fluid samples were routinely collected for microbiological culture at tube insertion, whereas some MT patients had already undergone diagnostic thoracentesis, antibiotic treatment, or prior drainage attempts before thoracoscopy and therefore did not always have repeat pleural fluid culture submitted at the time of MT. The positive culture rate was 17.8% in the TT group and 6.7% in the MT group, but this difference did not reach statistical significance (*P* = .259). Importantly, although pleural biopsies were obtained during MT in most cases, these were not routinely sent for microbiological culture, which may partially explain the low culture positivity rate in the MT group (see Table 2).

**Table 2:** Comparison of laboratory findings between groups.

Laboratory item	Tube thoracostomy group (n = 54)	Medical thoracoscopy group (n = 25)	Statistic	*P* value
Blood tests				
WBC (×10^9^/L)	11.5 (7.9–17.0)*	13.2 (9.5–21.7)*	*Z* = −1.075	.282
Neutrophils (%)	83.5 (76.0–87.4)*	83.4 (72.4–93.3)*	*Z* = −0.712	.477
ESR (mm/h)	58.9 ± 28.3†	60.9 ± 27.0†	*t* = −0.298	.766
hs-CRP (mg/L)	138.4 ± 70.4†	132.3 ± 65.5†	*t* = 0.365	.716
Pleural fluid tests				
WBC (×10^6^/L)	14,400.0 (1887.5–100,000.0)*	5211.0 (2316.5–15,650.0)*	*Z* = −1.256	.209
Monocytes (%)	10.0 (10.0–16.3)*	10.0 (10.0–11.1)*	*Z* = −0.765	.444
Neutrophils (%)	90.0 (83.8–90.0)*	90.0 (89.0–90.0)*	*Z* = −0.765	.444
LDH (U/L)	3905.0 (1428.5–11,943.5)*	3952.0 (1170.0–6870.5)*	*Z* = −0.432	.666
Albumin (g/L)	39.8 (22.6–50.9)*	42.9 (37.6–50.0)*	*Z* = −1.107	.268
Glucose (mmol/L)	0.8 (0.02–4.27)*	1.2 (0.04–4.37)*	*Z* = −0.143	.887
Pleural fluid culture				
Culture submitted	45 (83.3%)	15 (60.0%)	χ^2^ = 5.093	.024*
Culture positive	8 (17.8%)	1 (6.7%)	–	.259

* Median (IQR).

† Mean ± standard deviation.

* *P* < .05.

### 3.3. Clinical outcomes

The median total hospital stay was 17 days (IQR, 13–22) in the TT group and 20 days (IQR, 16–25) in the MT group, with no statistically significant difference (*P* = .149; 95% CI for median difference: −2 to 6 days).

The median pre-intervention hospital stay was significantly longer in the MT group (7 days, IQR 5–11) than in the TT group (2 days, IQR 1–3) (*P* < .001; 95% CI for median difference: 3–6 days). Conversely, the median post-intervention hospital stay was significantly shorter in the MT group (10 days, IQR 6–17) than in the TT group (15 days, IQR 11–20) (*P* = .043; 95% CI for median difference: −8 to 0 days).

The median total volume of pleural fluid drained was significantly greater in the MT group (1210 mL, IQR 475–2300) than in the TT group (600 mL, IQR 245–1212) (*P* = .016; 95% CI for median difference: 100–1000 mL).

There were no significant differences in clinical improvement rate (TT: 87.0% vs MT: 88.0%; difference: 1.0%, 95% CI: −15.2% to 17.2%, *P* = .901), treatment failure rate (TT: 5.6% vs MT: 12.0%; difference: 6.4%, 95% CI: −7.3% to 20.1%, *P* = .376), or in-hospital mortality (TT: 7.4% vs MT: 0%; difference: −7.4%, 95% CI: −17.1% to 2.3%, *P* = .317) between the TT and MT groups, respectively (see Table 3).

**Table 3:** Comparison of clinical outcomes before and after intervention.

Item	Tube thoracostomy group (n = 54)	Medical thoracoscopy group (n = 25)	Statistic (*Z*)	*P* value
Total hospital stay (d)	17 (13–22)*	20 (16–25)*	−1.441	.149
Pre-intervention stay (d)	2 (1–3)*	7 (5–11)*	−5.504	<.001*
Post-intervention stay (d)	15 (11–20)*	10 (6–17)*	−2.022	.043*
Pre-intervention fever (d)	9 (5–16)*	12 (8–19)*	−0.848	.396
Post-intervention fever (d)	1 (0–3)*	1 (0–3)*	−0.470	.638
Total pleural fluid drained (mL)	600 (245–1212)*	1210 (475–2300)*	−2.398	.016*
Improvement rate	87.0% (47)	88.0% (22)	–	1.000
Treatment failure rate	5.6% (3)	12.0% (3)	–	.374
In-hospital mortality	7.4% (4)	0% (0)	–	.302

* Median (IQR).

* *P* < .05.

### 3.4. Adverse reactions

In the TT group, adverse reactions included subcutaneous emphysema (1.8%) and tube-site swelling (1.8%). In the MT group, adverse reactions included subcutaneous emphysema (8.0%), re-expansion pulmonary edema (4.0%), and transient hypotension (4.0%). The subcutaneous emphysema cases were managed conservatively with continued drainage and observation; the re-expansion pulmonary edema improved with oxygen therapy, temporary adjustment of drainage, and supportive care; and transient hypotension responded to fluid support and monitoring. No procedure-related death or persistent serious sequelae occurred. The overall adverse reaction rate was 3.6% in the TT group and 16.0% in the MT group, with no statistically significant difference (difference: 12.4%, 95% CI: −2.1% to 26.9%, *P* = .076) (see Table 4).

**Table 4:** Comparison of post-intervention adverse reactions.

Adverse reaction	Tube thoracostomy group (n = 54)	Medical thoracoscopy group (n = 25)	χ^2^	*P* value
Hypotension	0	1 (4.0%)	–	–
Tube-site swelling	1 (1.8%)	0	–	–
Subcutaneous emphysema	1 (1.8%)	2 (8.0%)	–	–
Re-expansion pulmonary edema	0	1 (4.0%)	–	–
Total	2 (3.6%)	4 (16.0%)	–	.076

## 4. Discussion

The incidence of PPE among hospitalized patients with lower respiratory tract infections is substantial, with a minority progressing to CPPE or empyema.^[3]^ Mortality rates for PPE and empyema can reach 30%.^[7–9]^ Primary treatments are antibiotics and chest tube drainage. Drainage fails in about 30% of patients due to loculation and fluid viscosity, and about 20% require surgery.^[14,16]^ Due to a lack of large-scale Randomized Controlled Trial evidence,^[18]^ the role of MT in managing PPE/empyema lacks clear consensus. Recent guidelines and reviews continue to emphasize timely antibiotics, image-guided drainage, intrapleural enzyme therapy, and surgical escalation when drainage fails, while noting that the optimal position of MT in the treatment pathway remains insufficiently defined.^[5,22]^ This retrospective study compared the efficacy and safety of MT versus TT for CPPE/empyema.

### 4.1. Efficacy of MT

The improvement rate was 88% in the MT group and 87% in the TT group, with no significant difference. A meta-analysis by Mondoni et al^[21]^ reported MT success rates of 75% to 94% (pooled 85%) for empyema, consistent with our findings, suggesting MT is an effective modality. The 87% efficacy rate in our TT group is higher than the 66% reported by Metin et al^[23]^ and contrasts with a 37% failure rate reported by Mandal et al.^[24]^ Possible reasons include: intrapleural urokinase (used in 22.2% of our TT group) and saline irrigation may have improved success, as fibrinolytics increase treatment success^[25–27]^; manual saline lavage has also shown benefit^[28]^; variations in definitions of “success” across studies; and differences in patient inclusion criteria and disease severity. As highlighted in recent reviews, success in pleural infection management depends on timely diagnosis, appropriate antibiotics, and effective drainage.^[22]^

### 4.2. MT and post-intervention hospital stay

Post-intervention hospitalization was significantly shorter in the MT group (10 vs 15 days). A prospective RCT by Kheir et al^[29]^ also found that early MT shortened hospitalization compared to intrapleural fibrinolytic therapy. This may be because MT allows direct mechanical lysis of adhesions and optimal chest tube placement, accelerating fluid drainage more effectively than chemical fibrinolysis. The significantly greater drainage volume in the MT group (1210 vs 600 mL) supports this.

However, an important alternative explanation for the shorter post-intervention stay in the MT group warrants consideration. Because MT was performed later in the hospital course (median 7 vs 2 days for TT), patients in the MT group may have already begun to stabilize on antibiotic therapy, potentially biasing the post-intervention recovery period. This confounding by indication is inherent to retrospective observational studies and precludes definitive conclusions about procedural superiority. Total hospital stay did not differ significantly (20 vs 17 days), consistent with Kheir et al,^[29]^ and the prolonged pre-intervention time in the MT group (7 vs 2 days) – possibly due to preoperative preparation or delayed escalation from TT – may explain the lack of difference in total stay. A recent feasibility RCT by Bedawi et al^[11]^ compared early VATS with intrapleural enzyme therapy and highlighted the need for further research to define the optimal interventional strategy in pleural infection.

### 4.3. Bacterial culture positivity rate

Pleural fluid culture positivity was lower in our study (MT: 6.7%, TT: 17.8%) compared to others (e.g., 42% in Metin et al,^[23]^ 58.5% in Ravaglia et al^[30]^). This likely reflects 2 factors: the lower culture submission rate in the MT group (60% vs 83%) and the fact that pleural biopsies obtained during MT were not routinely sent for microbiological culture in our study. Previous studies have demonstrated that culturing pleural biopsy specimens can increase microbiological yield by up to 20% in pleural infection.^[31]^ A recent cohort study by Ravaglia et al^[32]^ further emphasized the importance of early intervention in improving outcomes. Future studies should ensure that both pleural fluid and biopsy specimens are routinely submitted for culture to optimize microbiological diagnosis and guide targeted antibiotic therapy.

### 4.4. Timing of MT intervention in pleural infection

Management requires antibiotics and early, effective drainage to clear pus.^[33]^ For failed conservative drainage, guidelines accept intrapleural fibrinolytics, VATS, or thoracotomy.^[34]^ MT, as a minimally invasive procedure between TT and VATS, can mechanically break loculations, lyse adhesions, facilitate drainage/lavage, and allow guided tube placement and pleural biopsy. Retrospective studies suggest better outcomes with early MT.^[35]^ It can be performed under local anesthesia/sedation, making it suitable for frail patients. Our and other observational studies suggest MT may be safe and effective, potentially shortening hospital stay,^[28,36]^ though a minority (approximately 7%) may require conversion to VATS/open surgery.^[3]^ In our cohort, the MT group had a longer pre-intervention period, suggesting that MT was often used after initial stabilization or delayed escalation rather than as immediate 1st-line drainage. This timing issue may partly explain why the post-intervention stay was shorter, but total hospital stay was not significantly reduced.

### 4.5. Complications

Postoperative complications occurred in 16% of the MT group and 3.6% of the TT group, although the difference was not statistically significant. In the MT group, complications consisted of subcutaneous emphysema in 2 patients, re-expansion pulmonary edema in 1 patient, and transient hypotension in 1 patient. Subcutaneous emphysema likely reflected air leakage through the pleural access tract or around the chest tube after adhesiolysis; both cases resolved with conservative management and continued drainage. Re-expansion pulmonary edema occurred after the evacuation of a large pleural collection and was managed with oxygen therapy, drainage adjustment, and supportive care. Transient hypotension occurred during or soon after the procedure and improved with fluid support and close monitoring. In the TT group, complications included 1 case of subcutaneous emphysema and 1 case of tube-site swelling, both of which were mild. These events are consistent with known procedure-related risks of pleural interventions. No procedure-related mortality or persistent serious sequelae occurred, supporting the procedural feasibility of MT in selected patients; however, the numerically higher adverse reaction rate in the MT group should be interpreted cautiously because of the small sample size and greater baseline disease complexity.

### 4.6. Limitations

This study has several important limitations that must be acknowledged when interpreting the findings. First, the retrospective design introduces inherent biases, including selection bias and confounding by indication, as treatment allocation was not randomized and was non-blinded, based on physician discretion, imaging findings, clinical status, and procedural suitability. Second, this was a single-center study conducted at a tertiary referral hospital, which may limit the generalizability of our findings to other settings with different patient populations or practice patterns. Third, the sample size was relatively small (n = 79), particularly in the MT group (n = 25), which limits statistical power to detect differences in rare outcomes such as mortality and increases the risk of type II error. Fourth, the 2 groups were imbalanced in size (TT n = 54 vs MT n = 25), reflecting real-world treatment selection rather than random allocation. Fifth, due to the small sample size and exploratory nature of the study, we did not perform multivariate adjustments for potential confounders, despite baseline imbalances between groups. Sixth, there is potential for unmeasured confounding from variables not captured in the medical records, such as detailed nutritional status, frailty indices, or specific antibiotic selection and duration. Seventh, pleural biopsy specimens obtained during MT were not routinely submitted for microbiological culture, which may have reduced microbiological yield in the MT group. Finally, the lack of blinding of outcome assessors, while unavoidable in a retrospective study, represents an additional source of potential bias. Therefore, these findings should be considered hypothesis-generating rather than definitive, and confirmation in prospective randomized trials is essential.

## 5. Conclusion

In conclusion, this retrospective study suggests that medical thoracoscopy is a feasible and safe minimally invasive technique for the treatment of complicated parapneumonic effusion and empyema in selected patients. Although MT was associated with more effective pleural drainage and shorter post-intervention hospitalization compared to tube thoracostomy, despite being performed in patients with more complex disease, it did not show a statistically significant effect on in-hospital mortality. Given the observational nature of this study, non-blinded treatment allocation, inherent selection bias, and small sample size, these findings should be considered hypothesis-generating rather than definitive. The procedure appears to have an acceptable safety profile, but the optimal timing of MT intervention and its comparative effectiveness relative to other treatment modalities remain to be established. Large-scale, multicenter randomized controlled trials are urgently needed to determine the precise role of medical thoracoscopy in the management pathway for pleural infection.

## Acknowledgments

The authors thank the patients and clinical staff involved in this study.

## Author contributions

**Conceptualization:** Yanchao Liu, Xin Liu, Zheng Duan.

**Data curation:** Yanchao Liu, Yaojie Wang, Xin Liu, Zheng Duan.

**Formal analysis:** Yanchao Liu.

**Funding acquisition:** Yanchao Liu.

**Investigation:** Yanchao Liu.

**Methodology:** Yanchao Liu, Yaojie Wang, Xin Liu.

**Project administration:** Yanchao Liu, Zheng Duan.

**Resources:** Yanchao Liu, Zheng Duan.

**Software:** Yanchao Liu.

**Supervision:** Yanchao Liu, Yaojie Wang, Xin Liu.

**Validation:** Yanchao Liu.

**Visualization:** Yanchao Liu.

**Writing – original draft:** Yanchao Liu, Yaojie Wang, Zheng Duan.

**Writing – review & editing:** Yanchao Liu, Zheng Duan.
